# A systematic review and meta-analysis of robot-assisted versus laparoscopically assisted gastrectomy for gastric cancer

**DOI:** 10.1097/MD.0000000000008797

**Published:** 2017-12-01

**Authors:** Yi Wang, Xudong Zhao, Yanjing Song, Aizhen Cai, Hongqing Xi, Lin Chen

**Affiliations:** Department of General Surgery, Chinese People's Liberation Army General Hospital, Beijing, China.

**Keywords:** gastric cancer, laparoscopically assisted gastrectomy, meta-analysis, robot-assisted gastrectomy

## Abstract

**Background:**

Robotic-assisted gastrectomy (RAG) has been used for gastric cancer since 2002. This meta-analysis was carried out to evaluate whether RAG is safer and more effective than conventional laparoscopically assisted gastrectomy (LAG) for gastric cancer.

**Methods:**

We performed a manual search for these 2 types of operations (RAG and LAG) in the PubMed, Embase, and the Cochrane Library databases up to April 30, 2016. Twelve nonrandomized controlled trials that reported on RAG and LAG for gastric cancer were included. Outcomes evaluated included operation time, number of retrieved lymph nodes, blood loss, length of the resection margin, complications, and postoperative hospital stay.

**Results:**

A total of 3744 patients in 12 studies were included (1134 patients in the RAG group and 2610 patients in the LAG group). The operation time was significantly shorter in the LAG group [weighted mean difference (WMD) 42.0 (95% confidence interval, 95% CI 28.11–55.89) minutes; *P* < .00001], while the loss of blood volume was lower in the RAG group (*P* = .01). The number of retrieved lymph nodes, duration of postoperative stay, length of the proximal resection margin, length of the distal resection margin, and postoperative complications were similar between groups.

**Conclusion:**

We conclude that RAG is a safe and appropriate treatment for gastric cancer patients in comparison to LAG. Nevertheless, RAG is not superior to LAG. Future research on RAG should focus on comparing the differences in retrieved lymph nodes in different tiers, evaluating the postoperative recovery and reducing the cost of the treatment.

## Abstract 2

Supplemental Digital Content is available in the text

## 1 Introduction

Laparoscopic surgery has been the predominant minimally invasive surgical approach since the 20th century.^[1]^ This approach has many advantages compared with traditional open surgery, such as a shorter hospital stay; decreased postoperative pain; earlier flatus, walking and intake; and less invasiveness.^[2]^ The first laparoscopically assisted Billroth I gastrectomy for early gastric cancer was reported in 1994.^[3]^ In the 2 decades that followed, laparoscopically assisted gastrectomy (LAG) was widely adopted in East Asia and mainly performed for patients with early gastric cancer, especially for stage I cancers.^[4–8]^ However, LAG is still controversial for > stage II cancers and suffers from technical limitations. For instance, the video camera is unstable, straight laparoscopic instruments have limited motion in a confined space, and surgeons must complete the operation with 2-dimensional images. Robotic surgery offers preliminary solutions to most of these problems. The da Vinci Surgical System provides a 3-dimensional, magnified view of the operating field, restores the natural hand-eye coordination, and offers more flexibility through its surgical instruments.^[9]^ The world's first da Vinci robot-assisted radical gastrectomy was completed in 2002.^[10]^ Since then, several studies have demonstrated the safety and efficacy of robotic surgical systems in gastric cancer.^[11–13]^ However, these studies included small sample sizes and had a single-factor design. The disadvantages of robotic surgery have been mentioned. The cost increases significantly, which has become an obstacle for robotics to spread in developing countries. Surgeons should accept training to use the new systems, but we still had no unified standard to complete the necessary training and appraisals. Thus, we sought to perform a meta-analysis of available data to effectively compare robot-assisted gastrectomy (RAG) with LAG in gastric cancer.

## 2 Methods

### 2.1 Study selection

We systematically searched the PubMed, Embase, and Cochrane Library databases using the terms “stomach,” “gastric,” “cancer,” “carcinoma,” “robot,” “robotic,” “laparoscopy,” “laparoscopic,” “surgery,” “gastrectomy,” and “gastric resection.” All articles were published in English between September 2002 and April 2016 and included comparisons between RAG and LAG. The randomized controlled trials (RCTs) have priority.

### 2.2 Inclusion criteria

Studies were included that fulfilled the following criteria: clinical research comparing RAG with LAG for gastric carcinoma patients; full-text article containing necessary data for statistical analysis; and at least one of the following outcome measures (operation time, number of retrieved lymph nodes, estimated blood loss, duration of hospital stay, and postoperative complications). If 2 or more studies were reported by the same writer or center, we included the most recent publication or the higher quality publication. If 2 or more studies included totally different patients from the same center, we still analyzed the datum from those studies.

### 2.3 Exclusion criteria

Studies were excluded if they fulfilled the following criteria: reviews, conference reports, and case reports; studies without control groups; studies performing gastric surgery for gastrointestinal stromal tumors and bariatric surgery; studies without necessary data for statistical analysis; and duplicate research based on author or center.

### 2.4 Data analysis

We used the software Review Manager version 5.3 (Nordic Cochrane Centre, Copenhagen, Denmark) to analyze the selected studies. Only 3 or more than 3 studies could be analyzed simultaneously. Continuous variables were evaluated to obtain the weighted mean difference (WMD), and dichotomous variables were examined using the odds ratio (OR). The 95% confidence intervals (CIs) were established, and values of *P* < .05 were considered to indicate statistical significance. Publication bias was quantitatively evaluated using funnel plots. Statistical heterogeneity was evaluated by the Higgins *I*^2^ statistic. If *I*^2^ values were more than 50% and *P* < .10, it indicated the high heterogeneity so that we analyzed data with a random-effects model. Otherwise, we analyzed data with a fixed-effect model.

### 2.5 Ethical consideration

The IRB of Chinese PLA General Hospital approved our study.

## 3 Results

### 3.1 Selected studies

**Figure 1:**
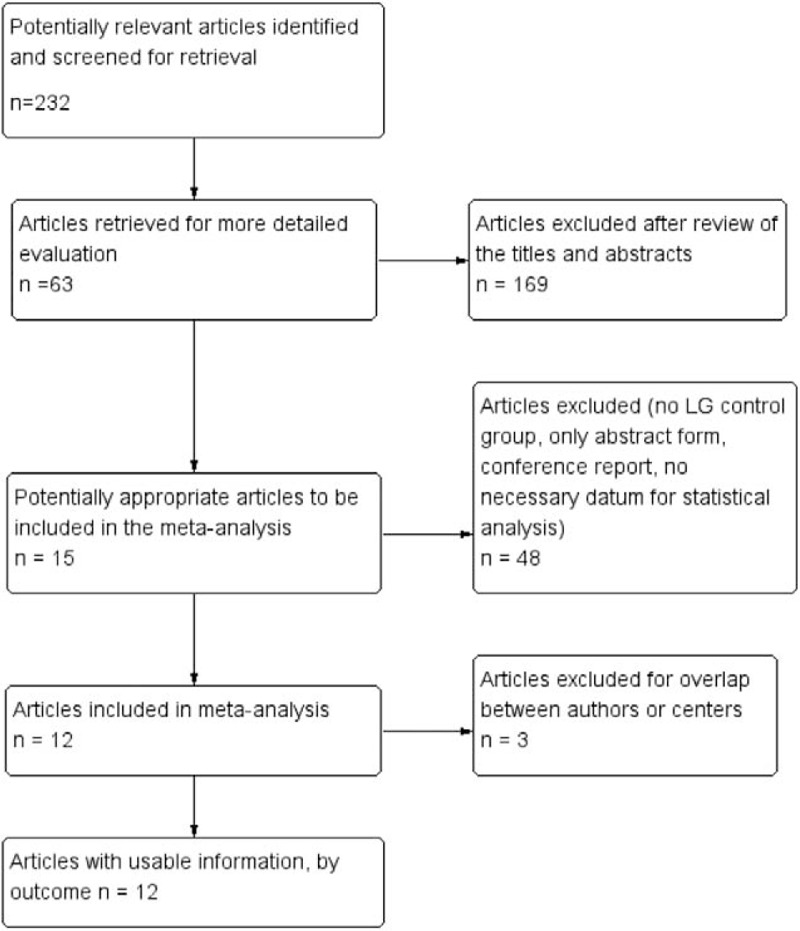
PRISMA diagram showing the selection of articles for review.

Figure 1 shows a flow diagram of our search strategy. The initial search of our databases identified 232 studies. Reviewers excluded 169 articles after a combined review of titles and abstracts. Studies were removed if they had no control group or only provided an abstract. Conference reports were also excluded. Finally, we obtained 12 articles with a total of 3744 patients for inclusion.^[12,14–24]^ Approximately 1134 patients were in the RAG group, while 2610 patients were in the LAG group. Kim et al,^[16]^ Kim et al,^[14]^ and Huang et al^[20]^ also reported open gastrectomy results, but we only analyzed data regarding RAG and LAG. Two studies^[15,17]^ came from the same center, but totally different patients were respectively included and performed different operations in each study. We obtained some unpublished data from four authors. All trials were observational studies (Table 1), and the methodological quality of these studies was assessed using a methodological index for nonrandomized studies (MINORS).^[25]^ The median quality score was 11.7 (Table 2).

**Table 1:**
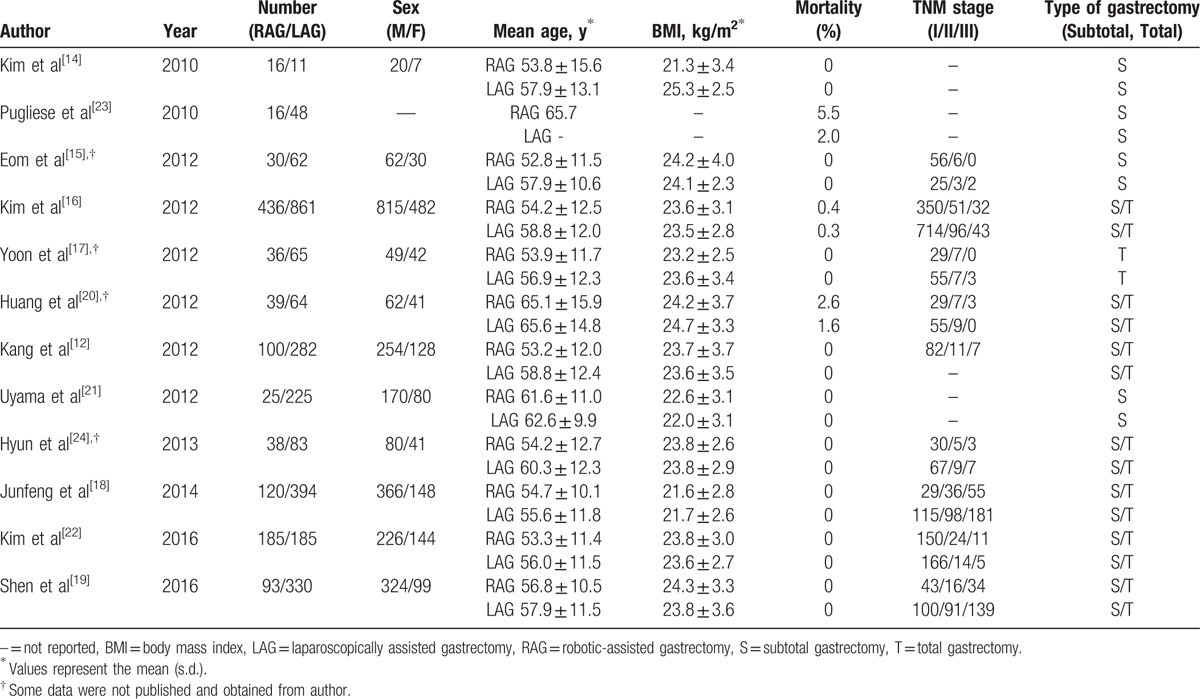
Characteristics of the 6 selected studies.

**Table 2:**
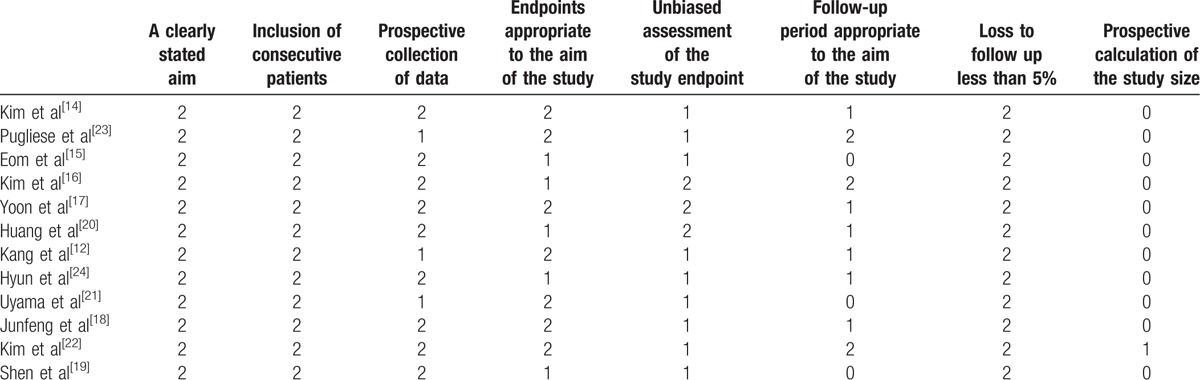
Assessment of the quality of the studies using the methodological index for nonrandomized studies (MINORS).

### 3.2 Outcome measure

**Figure 2:**
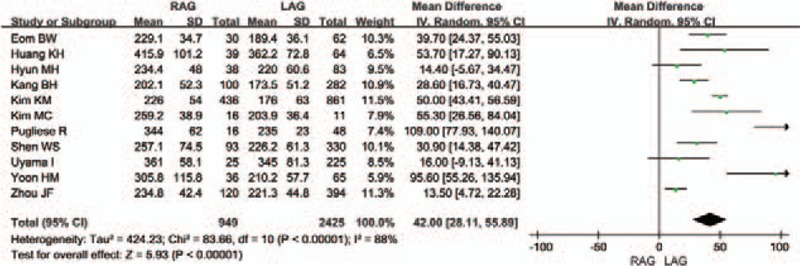
Forest plot comparing the operation time for RAG versus LAG.

All selected studies provided the duration of gastric cancer surgery. The meta-analysis showed that the operation time was clearly longer in the RAG group than in the LAG group [WMD 42.0 (95% CI 28.11–55.89) minutes; *P* < .00001]. The heterogeneity between studies was also significant (*I*^2^ = 88%) (Fig. 2).

**Figure 3:**
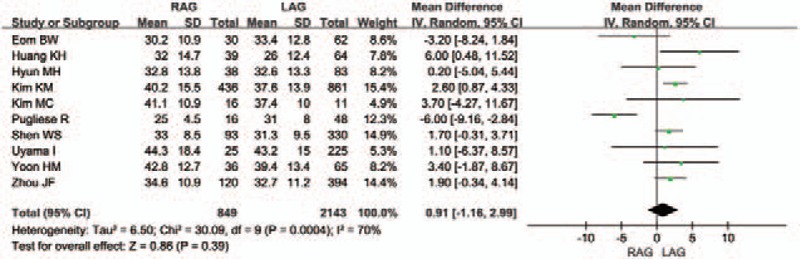
Forest plot comparing the number of retrieved lymph nodes for RAG versus LAG.

The mean number of retrieved lymph nodes was reported clearly in 11 articles. The variation in the number of harvested lymph nodes was significant (*I*^2^ = 70%), and there was no difference between the 2 groups [WMD 0.91 (95% CI -1.16 to 2.99); *P* = .39] (Fig. 3).

**Figure 4:**
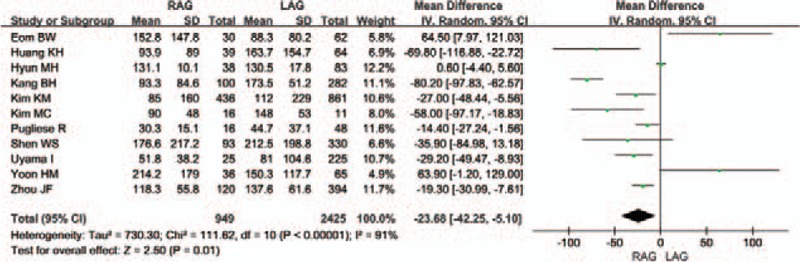
Forest plot comparing the volume of blood loss for RAG versus LAG.

The blood loss volume during surgery was reported with precise standard deviations. The meta-analysis showed that the mean blood loss volume was lower in the RAG group, and this result had obvious statistical significance [WMD -23.68 (95% CI -42.25 to -5.10) mL; *P* = .01]. The heterogeneity between studies was significant (*I*^2^ = 91%) (Fig. 4).

**Figure 5:**
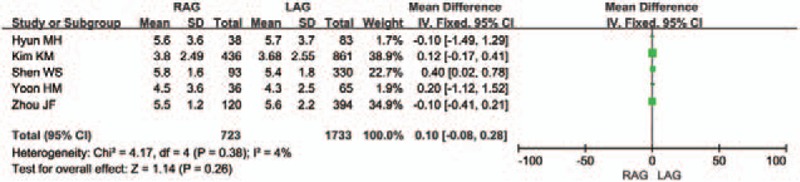
Forest plot comparing the length of the proximal resection margin for RAG versus LAG.

**Figure 6:**
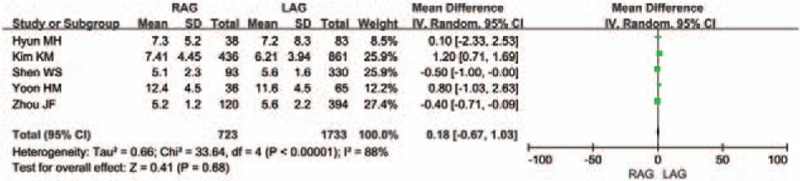
Forest plot comparing the length of the distal resection margin for RAG versus LAG.

Five of the 12 studies reported the length of the proximal resection margin and distal resection margin. Five of the remaining 7 studies^[12,14,20,21,23]^ did not report this variable, and the other 2 studies^[15,22]^ only showed incomplete data. There was no difference between the 2 groups in terms of the length of the proximal resection margin [WMD 0.1 (95% CI −0.08 to 0.28) cm; *P* = .26] (Fig. 5). The difference between the 2 groups in the length of the distal resection margin was also unconspicuous [WMD 0.18 (95% CI -0.67 to 1.03) cm; *P* = .68], but the heterogeneity was significant (*I*^2^ = 88%) (Fig. 6).

**Figure 7:**
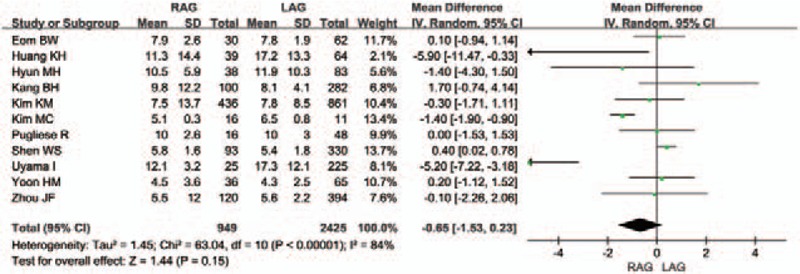
Forest plot comparing the duration of the postoperative hospital stay for RAG versus LAG.

All studies reported the duration of the postoperative hospital stay, and data of only 1 study^[22]^ could not be used to calculate the WMD (Fig. 7). The meta-analysis revealed significant heterogeneity between the RAG group and the LAG group (*I*^2^ = 84%), with the similar hospital stay after RAG and LAG [WMD -0.65 (95% CI -1.53 to 0.23) days; *P* = .15]. As the potential influencing factors of postoperative hospital stay, the days of oral intake and first flatus were analyzed, respectively. There was no difference between the 2 groups in either one of them (Supplemental Figure 1, 2).

**Figure 8:**
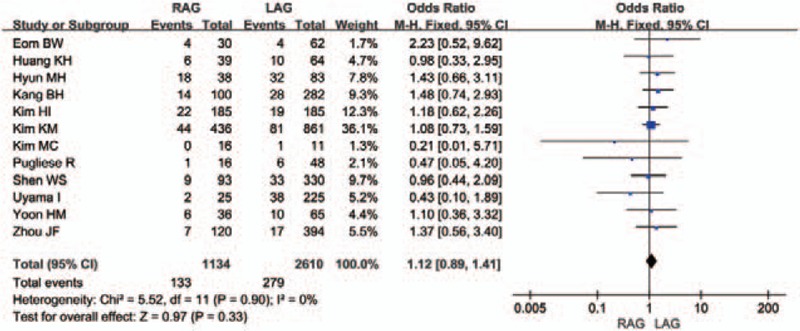
Forest plot comparing the early postoperative complications for RAG versus LAG.

Early postoperative complications were recorded in all studies (Fig. 8). The incidence of early postoperative complications was similar after RAG and LAG (OR 1.12, 95% CI 0.89–1.41; *P* = .33), without evidence of statistical heterogeneity (*I*^2^ = 0%). Eight studies^[12,16–20,22,24]^ classified details of the complications by subgroups in different standards, which included wound infection, anastomotic leakage, anastomotic stenosis, ileus and obstruction, fluid collection and bleeding, abscess, and so on. There were no differences in the risk of early postoperative complications between the 2 groups in all main subgroups, without evidence of statistical heterogeneity (Supplemental Figure 3–8).

**Figure 9:**
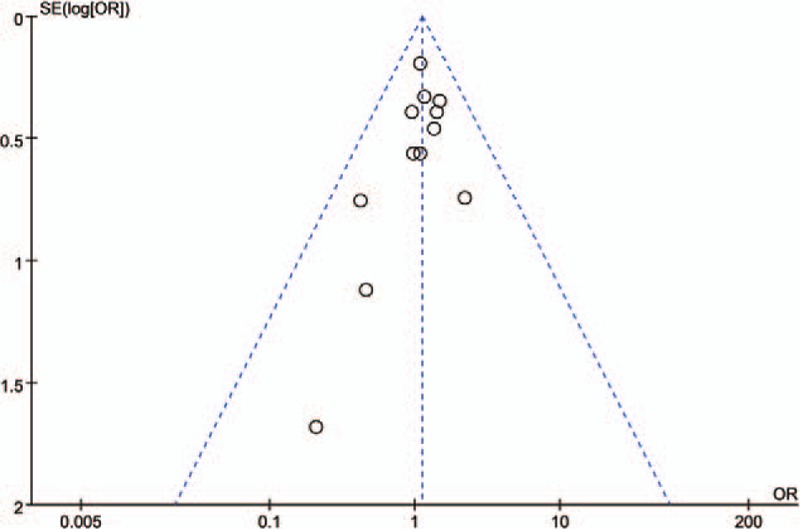
Funnel plot for results from all studies comparing the overall complication rate.

The funnel plot for the primary outcome of postoperative complications was relatively symmetrical between the 2 groups, suggesting that publication biases were not serious. All studies remained inside the limits of the 95% CI (Fig. 9).

## 4 Discussion

In East Asia, gastric cancer is one of the most common malignant diseases.^[26]^ Surgical resection is the preferred treatment for gastric cancer patients. With the development of minimally invasive techniques, minimally invasive surgery has been widely accepted as an important method for gastric cancer surgery. Many studies have compared the safety and short-term or long-term efficacy of LAG with open gastrectomy.^[5,6,27–30]^ However, studies on RAG have not been sufficient to evaluate the treatment's effectiveness, as most of these studies were not randomized and were based on a single center's experience. We selected 12 clinical trials and proceeded to complete a meta-analysis to investigate the safety and value of RAG for the treatment of gastric cancer.

Our meta-analysis revealed that surgeons spent more operative time in RAG than in LAG. The robotic surgery system usually includes the surgeons’ console, patient cart (surgical cart), and video cart (equipment cart). Before the operation, surgeons and nurses need to assemble the manipulator arms and debug the equipment, such that RAG requires extra time for preparation. However, this procedure often requires less than 30 minutes.^[31,32]^ In addition, the learning curve for RAG is an important factor influencing the time spent during surgery. Eom et al^[15]^ reported that operating times might stabilize after at least 15 cases and then shorten gradually, and surgeons who have experience in laparoscopic surgeries can reach a plateau in operating time after 20 cases.^[12,33]^ Some selected studies^[12,20,21]^ analyzed the operation time, blood loss, and duration of hospital stay in different phases (initial phase and late phase) in the RAG group. The results showed the importance of the surgeon's experience and the level of cooperation in the therapeutic team. If the operation team is stable and surgeons have enough experiences in laparoscopic surgeries (including subtotal and total gastrectomy), the operative time and volume of blood loss reduced significantly in the late phase. Another reason that could influence the operative time is the operation type. All of the selected studies illustrated the main types of gastrectomy, compared the differences between subtotal and total gastrectomy, and the results showed no statistical difference.

As prognostic factors of surgical therapy for tumors, the number of retrieved lymph nodes and resection margin cannot be ignored. This analysis showed that there was no obvious difference in the total number of lymph nodes resected between RAG and LAG. Robot-assisted surgery is based on laparoscopic surgery, and the operative steps of lymph node dissection are generally the same as those in LAG. In addition to the above reasons, so many gastric cancer patients showed stage I clinicopathology in our meta-analysis, and the number of metastatic lymph nodes among different tiers was correspondingly reduced. These findings may not reflect the advantages of the robotic surgical system. Some of the selected studies^[16–19]^ precisely reported the tumor resection margin. The length of the proximal and distal resection margin was undifferentiated between the groups overall. This finding showed RAG could accomplish the range of radical resection in LAG, and we still believe the distance from the resection margin to the lesion is of great significance for surgeons.

The mean estimated blood loss in RAG was less than in LAG in our meta-analysis. Although most selected studies did not mention the same standard used to calculate the quantity of blood loss, this result shows that robot-assisted surgery is a new minimally invasive technique with great potential for development. Because the manipulator arms of the robotic surgery system are more stable than a surgeon's hands, the improved dexterity of an internal articulated wrist provides greater flexibility in a narrow operative field. Furthermore, 3-dimensional images also help surgeons to visualize small vessels. All the above reasons may contribute to avoiding accidental damage and decreasing blood loss during the operation. One point that is not entirely satisfactory is that the amount of bleeding decrease may be negligible for surgeons.

The duration of the postoperative hospital stay for patients accepting RAG was the same as that of patients undergoing LAG. Although the average length of hospital stay was reduced by nearly a day when comparing the 2 groups, the difference did not reach statistical significance. Time to diet, mobilization, first flatus, and drainage are potential factors that should have an important impact on postoperative recovery. However, not all the factors were precisely described in all the selected studies. Finally, we chose 6 selected articles,^[14,17–19,21,23]^ which contained sufficient data to analyze the days of oral intake and first flatus respectively, and found these 2 potential factors could not induce the different postoperative hospital stay between the 2 groups. We considered that patients receiving RAG might not recover faster than those undergoing LAG only depending on more advanced surgical techniques.

This meta-analysis indicated that early postoperative complication rates were equivalent between RAG and LAG. The included studies compared a variety of complications, such as wound infection, anastomotic leakage, anastomotic stenosis, bleeding, fluid collection, ileus, and obstruction. We analyzed these data in subgroups and demonstrated that there was no difference between the 2 groups. Only Kim et al^[16]^ and Hyun et al^[24]^ used the Clavien–Dindo classification as a complication grading system.^[34]^ According to the result of the postoperative hospital stay and complication rates in 2 groups, we considered the patients could have the similar degree of comfortable feeling after the RAG or LAG.

As an important assessment indicator, the patients’ financial burden should not be ignored. We found that the cost of RAG is higher than LAG in some studies.^[22,35,36]^ It is an apparent obstacle to encourage surgeons to use the robotic system in surgeries. But this could be overcome by the generalization of robots and financial supplements from the national health insurance system in the future.

Our meta-analysis has some limitations. All 12 studies were clinical observational trials, and they were neither randomized nor double-blind. Most of the selected studies came from East Asia, and ethnic differences should be considered as a factor that may have caused selection bias. In addition, the heterogeneities of operation time, blood loss, number of retrieved lymph nodes, duration of postoperative stay, and length of the distal resection margin were all significant. This result can be mainly attributed to the selection bias. In most of Korean studies, we could see patients who accepted RAG were younger and richer than those receiving LAG. The difference of surgeon’ s experience might also influence the choice of surgical procedures. The number of RAG procedures was less than 40 in 7 studies, which might have affected the comparison and heterogeneity between RAG and LAG. Last, only 2 selected studies reported the follow-up results. They all compared 3-year survival rate and found no significantly difference between RAG and LAG. We could not make a meta-analysis on such few data.

## 5 Conclusion

We believe that RAG is a safe and comfortable treatment method for gastric cancer patients in comparison to LAG. Nevertheless, RAG is not superior to LAG. A prospective, randomized, controlled clinical trial should be designed and carried out, and future research should focus on comparing the differences in retrieved lymph nodes in different tiers, evaluating the postoperative recovery and reducing the treatment expense.

## Supplementary Material

Supplemental Digital Content
