# Supplementary material for: A systematic review and meta-analysis of robot-assisted versus laparoscopically assisted gastrectomy for gastric cancer
Source: Medicine (Baltimore). 2017 Dec 1;96(48):e8797. doi: 10.1097/MD.0000000000008797 (PMC5728759; doi:10.1097/MD.0000000000008797)

Supplemental Figure 1 Forest plot comparing the days of oral intake for RAG versus LAG.


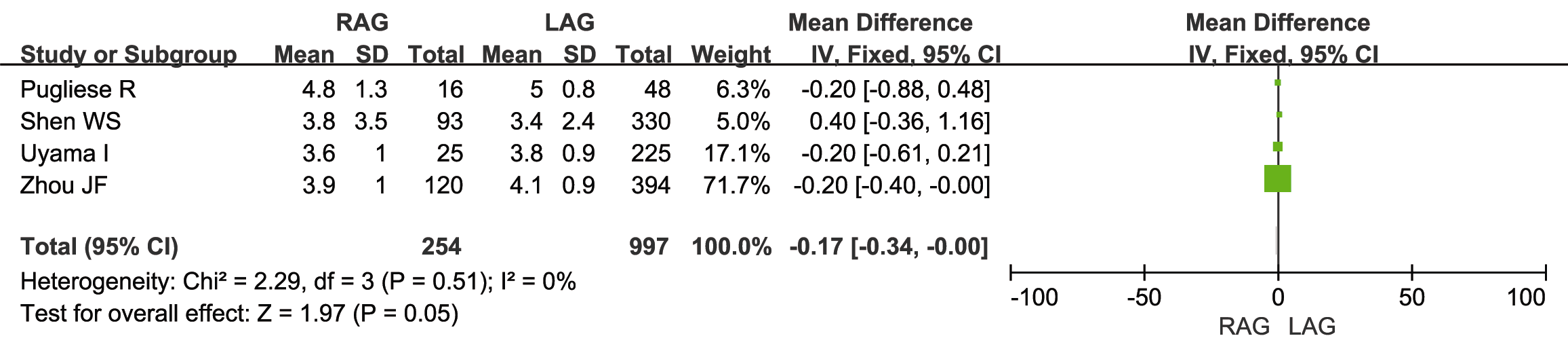


Supplemental Figure 2 Forest plot comparing the days of first flatus for RAG versus LAG.


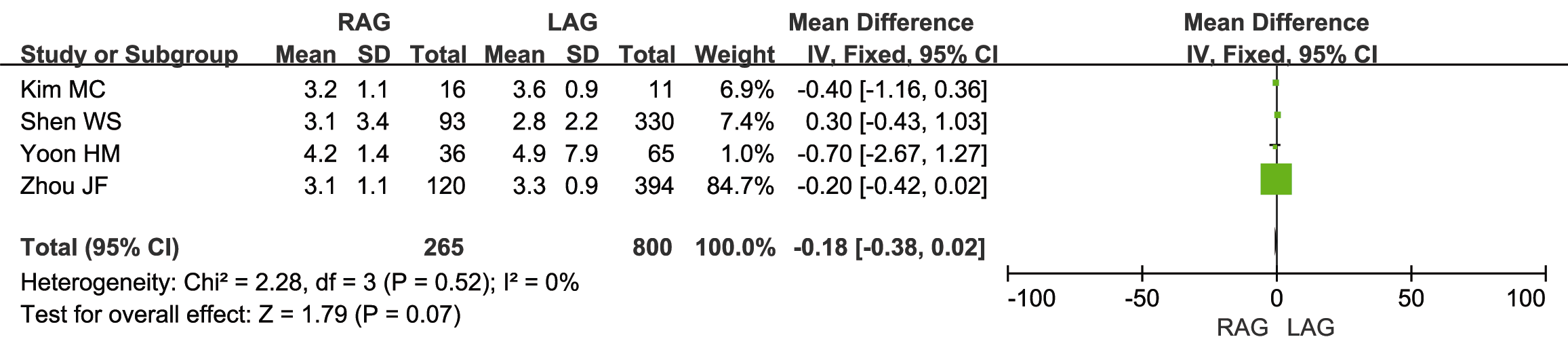


Supplemental Figure 3 Forest plot comparing the wound infection for RAG versus LAG.


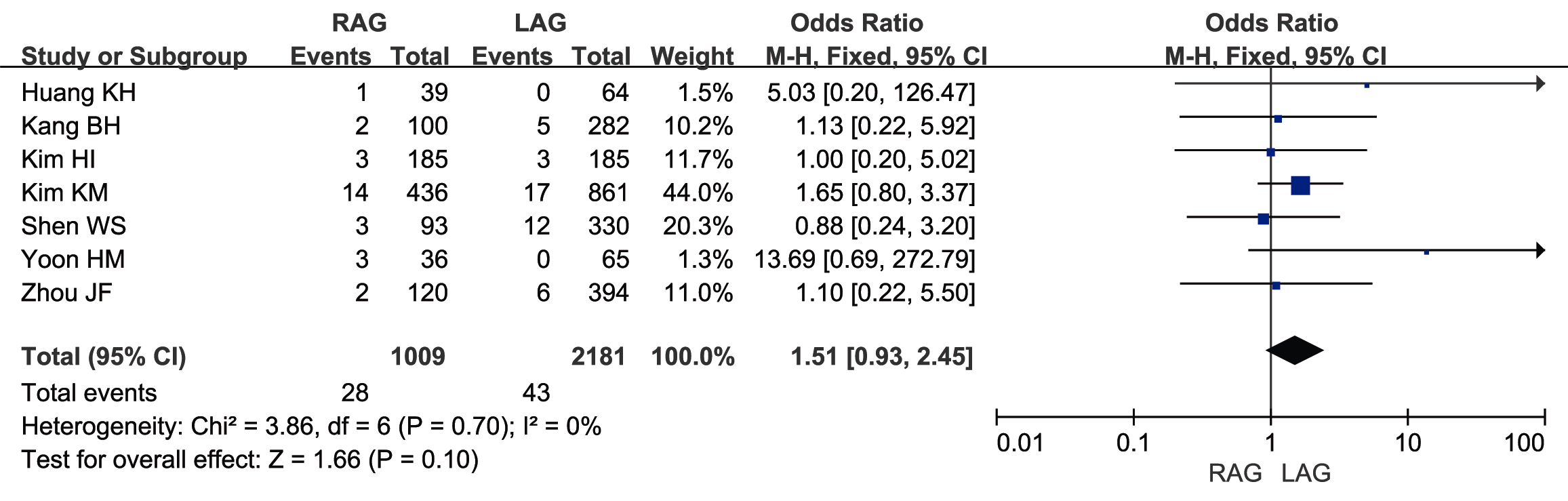


Supplemental Figure 4 Forest plot comparing the anastomotic leakage for RAG versus LAG.


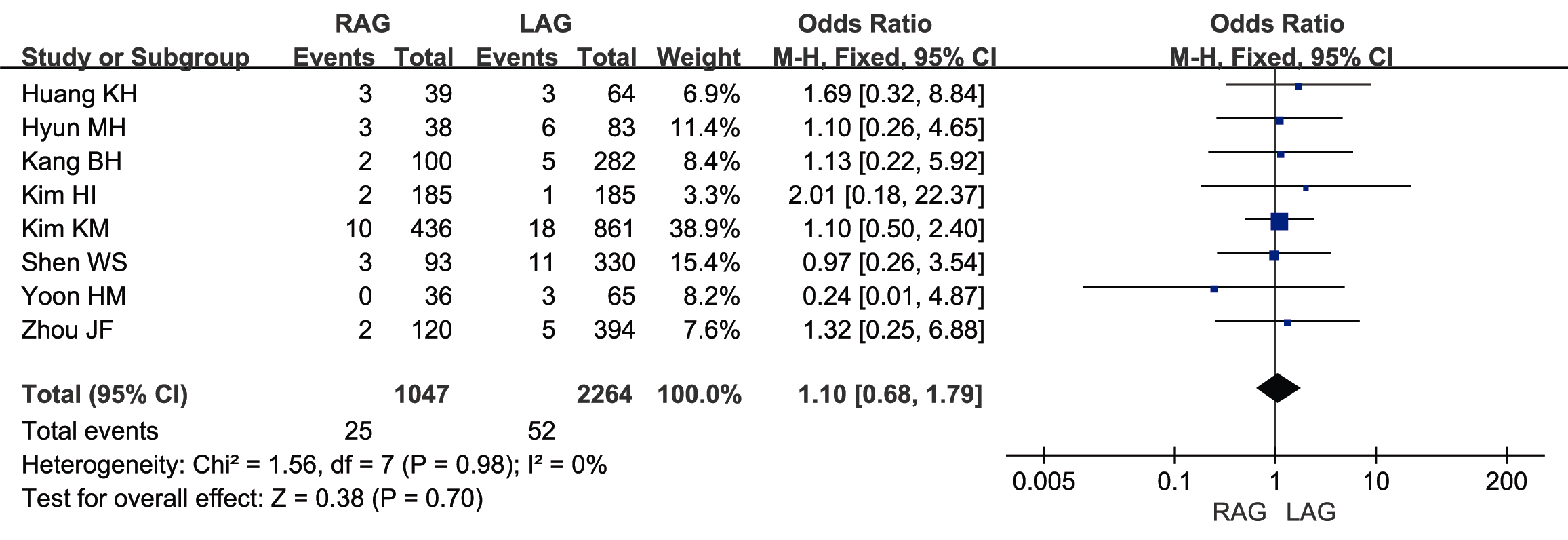


Supplemental Figure 5 Forest plot comparing the ileus and obstruction for RAG versus LAG.


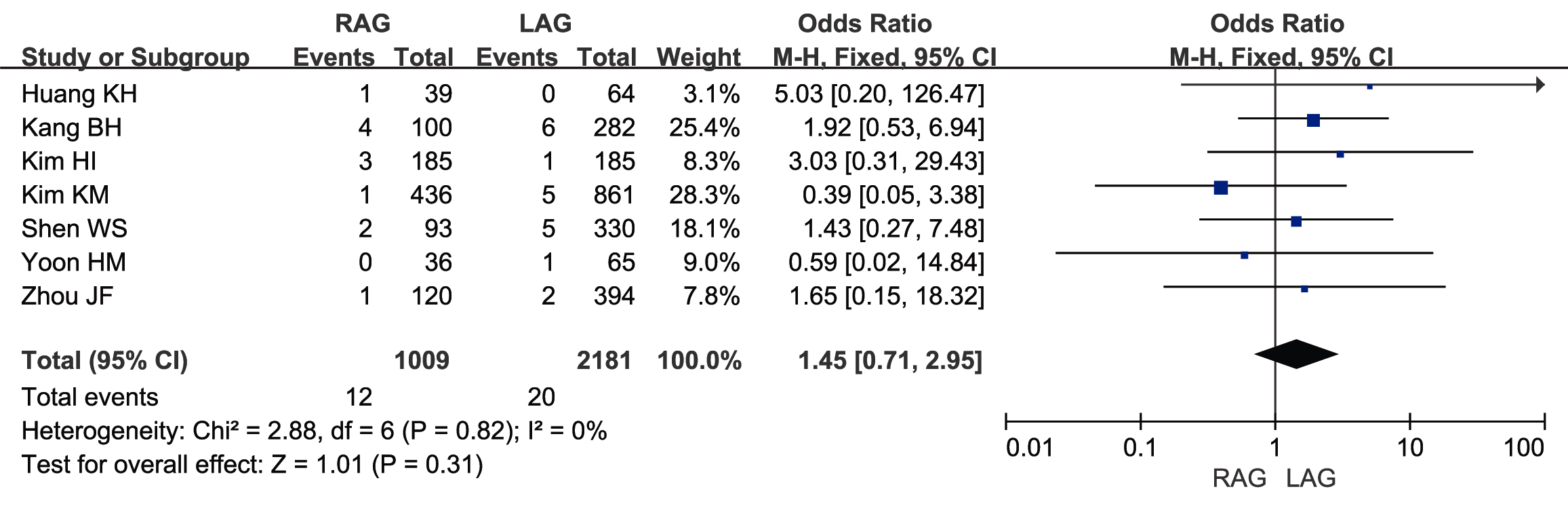


Supplemental Figure 6 Forest plot comparing the fluid collection for RAG versus LAG.


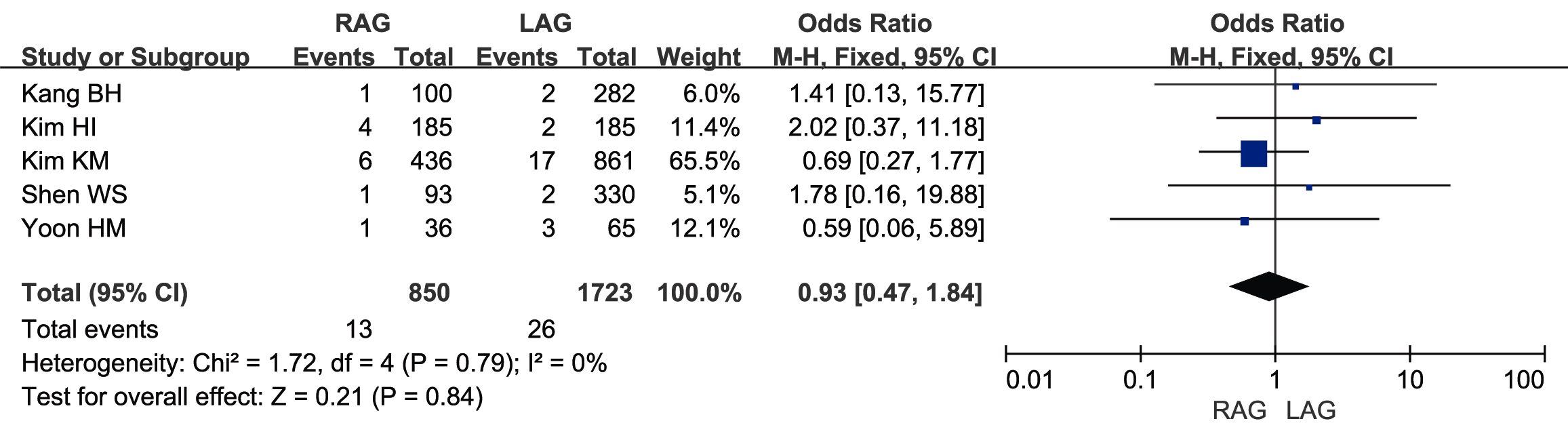


Supplemental Figure 7 Forest plot comparing the bleeding for RAG versus LAG.


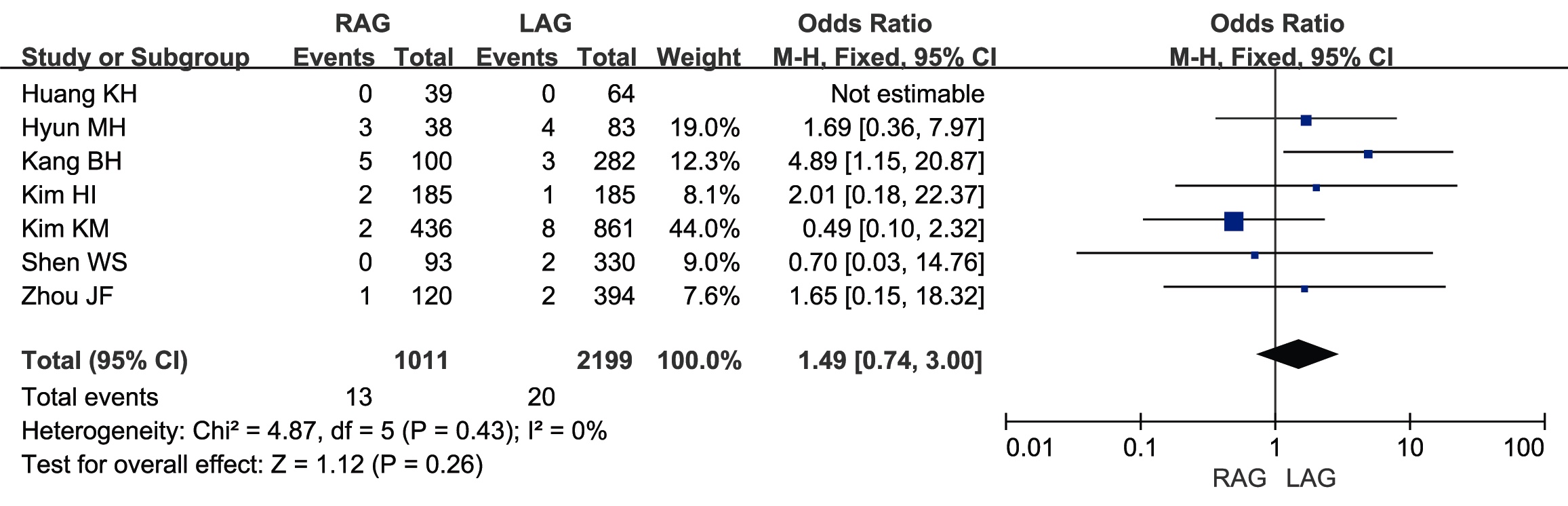


Supplemental Figure 8 Forest plot comparing the anastomotic stenosis for RAG versus LAG.


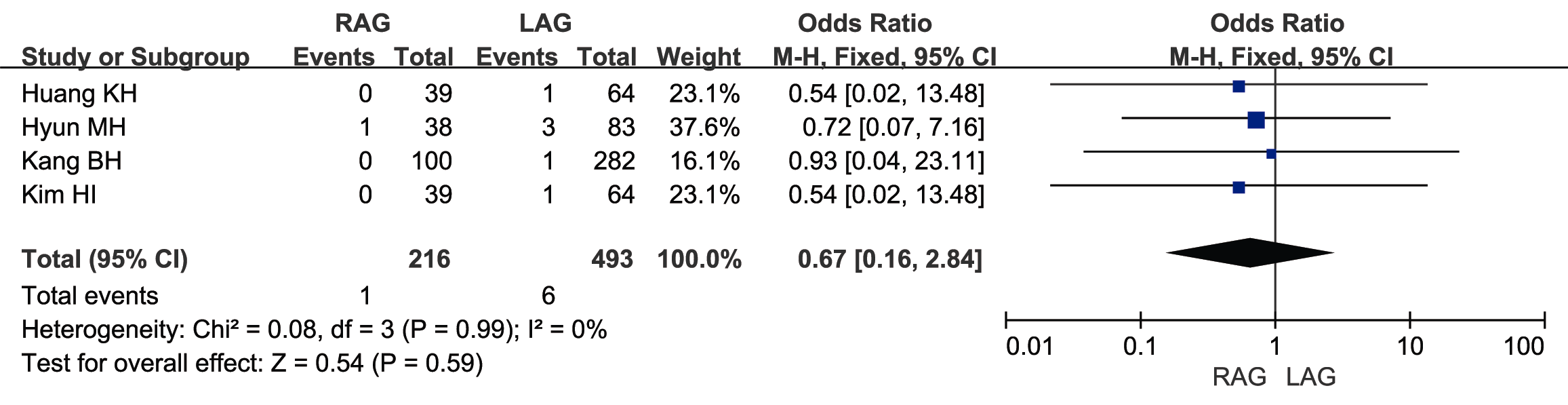

Supplement: Supplemental Digital Content [file medi-96-e8797-s001.doc]
